# Promoting older women’s mental health: Insights from Baby Boomers

**DOI:** 10.1371/journal.pone.0245186

**Published:** 2021-01-12

**Authors:** Maggie Kirkman, Jane Fisher

**Affiliations:** Global and Women’s Health, Public Health and Preventive Medicine, Monash University, Melbourne, Victoria, Australia; University of Calgary, CANADA

## Abstract

Optimal mental health underpins full social participation. As people age, they confront personal and cultural challenges, the effects of which on mental health are not fully understood. The aim of this research was to learn from women of the Baby Boomer generation (born 1946–1964) what contributes to and hinders their mental health and wellbeing. Eighteen women participated in qualitative interviews (in English); data were analysed thematically. Participants were located across Australia in rural and urban areas; not all were born in Australia. They were diverse in education, employment status, and experiences of life and ageing. The women nominated as the main contributors to poor mental health in older women *Illness and disability*, *Financial insecurity*, *Maltreatment*, and *Loss and grief*. Contributors to good mental health were identified as *Social interdependence*, *Feeling valued*, *Physical activity*, *Good nutrition*, and *Having faith or belief*. Women’s accounts supplied other influences on mental health, both associated with the person (*Personality* and *Intimate relationships and sex*) and with society (*Constructs of ageing*, *Gender*, and *Culture*). Women also specified what they needed from others in order to improve their mental health as they aged: *Public education about ageing*, *Purposeful roles for older women in society*, *Adequate services and resources*, and *Sensitive health care*. In sum, older women wanted to be treated with respect and for their lives to have meaning. It is evident from these results that circumstances throughout life can have profound influences on women’s mental health in older age. Anti-discriminatory policies, informed and inclusive health care, and social structures that support and enhance the lives of girls and women at all ages will therefore benefit older women and increase the potential for their continuing contribution to society. These conclusions have implications for policy and practice in well-resourced countries.

## Introduction

It is approaching cliché to note that the population is ageing. Too often, however, this is assumed to mean that we—as individuals and collectively—are in decline. This construction of older members of society can have an adverse effect on mental health (see the discussions in [1, 2]). People in the US aged at least 65 years who had one or more chronic health conditions revealed, a decade ago, that identity as an older person was influenced by stigma associated with ageing, whether or not the person identified as “old” [3]. A survey of adults aged 60 years and older and living in Australia found that having positive attitudes to one’s own ageing was associated with better physical and mental health, including lower levels of anxiety and depression [4]. The authors concluded that challenging negative stereotypes of ageing could contribute to healthier ageing [4]. In contrast to the negative stereotypes, the Australian Institute of Health and Welfare [5] reported that the increase in life expectancy found for the years 1998 to 2012 (four years for men, nearly three years for women) was matched by an increase in expected disability-free years.

The constructs and concomitants of ageing are complex. For example, recent anthropological work in the US explored the ways in which older people are expected to take steps to resist ageing [6]. Medical practice contributes to this endeavour, but “anti-old” attitudes and behaviour are class-related (people higher on the socioeconomic scale appear more committed to the moral imperative of youthfulness) and confined to the able-bodied [6]. A cross-cultural review of research published in English and Chinese (traditional characters) found that, in contrast with academic views, older lay people have a construct of healthy ageing that encompasses more than maintaining physical, mental, and social functioning [7]. Aspects of healthy ageing mentioned by lay people but not included in academic investigations were “family,” “adaptation,” “financial security,” “personal growth,” and “spirituality” [7]. This mismatch of lay and academic understanding is one reason for our consultation with older people themselves.

In addition to these cultural influences, there is evidence that the experience of ageing is gendered and that gender differences are multifaceted [8]. As they age, women were identified as being at a financial disadvantage in comparison with men [9]. There is no evidence that this discrepancy has been eradicated; older men in Australia were recently found to be financially more secure than older women [10]. Women claimed to have better overall wellbeing than men and gave a higher rating to their social interactions and their sense of connection to the community; they also tended to report spending more hours doing unpaid work than older men [10].

The effects on mental health of the challenges confronting people as they age in a well-resourced country such as Australia, as well as the contribution of good mental health to healthy ageing, are not fully understood. We employ the World Health Organization’s [11] definition of mental health: a state of wellbeing in which people can use their skills and capabilities, adapt to the demands of daily life, experience mutually gratifying relationships, and contribute to the community, including through productive work. It is much more than the absence of illness. Most research has focused on understanding severe or common mental disorders (see [12]). The factors that interact to foster good mental health, including in later life, are much less well elucidated.

Our trajectory analysis of data from the 1921–26 cohort of the Australian Longitudinal Study of Women’s Health (Waves 1–6) is the first investigation of mental health (rather than mental illness) in this large cohort of women who contributed comprehensive data from their early 70s until the end of their lives or at least the age of 85 years [12]. We identified three mental health trajectories: stable high (77%), stable low (18.2%), and declining from high to low (4.8%). We found that older women’s mental health is related to social relationships, general health, access to physical activity and healthy nutrition, coincidental adverse life events, and experiences of interpersonal violence. It is consistent with these results that a recent survey in the UK found that the extent to which people aged 50 and over assess their life as meaningful is associated with subsequent well-being, as measured on other variables [13].

Intimate partnerships are fundamental to human society; physical intimacy can be an important component of mental health [14, 15]. Nevertheless, Australian (and other) sexual health policies tend to exclude the possibility that older people are sexually active and need appropriate sexual health care, promotion, and prevention [16]. Analysis of a large data set in the Netherlands found that more than two thirds of partnered adults aged 65 and older engaged in physical tenderness and almost half were sexually active (as defined by participants) [17]. However, few unpartnered adults (and fewer women than men) reported either tenderness or sexual activity, suggesting that the greatest barrier to physical intimacy, especially for women, is the lack of a partner [17].

We began our investigation of what enhances and damages mental health by focusing on Baby Boomers. Members of this generation (born 1946–1964) are now in their fifties, sixties, and seventies. The cohort was identified early on as very different from the previous generation (known by demographers as the Silent Generation). Baby Boomers experienced, indeed contributed to, significant social change, including feminism and the sexual revolution [18, 19]. (Their contribution has not been seen as wholly benign: They have been represented as “selfish” and a “problem generation” [20].) Baby Boomers’ “old” age could differ from that of their predecessors, although this should not be assumed [21]. Baby Boomers do not constitute a homogeneous group. Interviews about sexuality with Baby Boomer (and older) women in Australia revealed diverse experiences and expectations [21]. Not all were ‘liberated’ in the ‘sixties and not all expected to be sexually active as they aged; some women identified the ‘sexual revolution’ as benefiting only men [21]. From the perspective of a transformative generation (whether members participated in, resisted, or observed that transformation), Baby Boomers can help us to understand how best to promote wellbeing, mental health, and the continuing contribution to society by women as they age.

Having optimal mental health is central to full social participation. It has long been argued that the process of ageing should be studied not just in relation to populations but from the perspective of older people themselves [8]. Our aim in this research was to learn from the Baby Boomer generation what they understand as contributing to and hindering their mental health and wellbeing. Because the experience of ageing is gendered, meaning that being female, male, or non-binary interacts with social and cultural norms and practices of ageing, we began by consulting women. Our research question, therefore, was: What do women of the Baby Boomer generation understand as contributing to and hindering their mental health and wellbeing? Our ultimate goal is to contribute to the improved mental health and wellbeing of older women in Australia and, potentially, other well-resourced countries.

## Method

We chose a qualitative interview design as the most appropriate means of seeking to understand participants’ perspectives on a topic [22]. We align ourselves with feminist epistemology [23], with a particular emphasis on feminist standpoint theory. Our approach is consistent with Interpretive Phenomenology [24], and this guides our analytic strategy. We sought not only to understand the perspective of each woman whose life experience and insights made her an expert knower in her own life, but also to find meaning in the ways in which women from a specific era made sense of their lives as a whole.

Eligibility requirements for participants were that they identified as women, were born in the years 1946–1964, lived in Australia, and could communicate in English. To recruit eligible women, we placed a notice about the research in the widely-distributed magazine (dated August 2018) of the not-for-profit organisation Jean Hailes Foundation (jeanhailes.org.au). We planned to recruit a maximum of about 30 women, with assessments of data quality to be made as interviews progressed so that rich but not excessive data could be collected. The maximum would have been sought only if interviews were brief, with sparse detail. There is evidence that about 12 interviews will yield adequate data [25]. There was no expectation that “saturation” of any kind would be achieved for such a complex life experience. Saturation is not a straightforward concept [26]; we did not assume that there is an ‘essence’ of the experience of ageing to be discovered, nor that we would exhaust the possibilities for nuanced variation in the accounts from older women. When women expressed interest in the research, we sent them a copy of the Explanatory Statement and an Informed Consent Form.

We accepted the first few women who agreed to participate. As recruitment progressed, we assessed characteristics such as age, education, relationship status (in a relationship or not, sex of partner), ethnicity, health status, and location (regional, rural, metropolitan) and selected subsequent participants for maximum diversity. We were aware that complete diversity and inclusion were unlikely to be achieved within the practical constraints of the project; for example, we were not funded to employ interpreters for women not fluent in English nor to travel for interviews.

We developed a study-specific semi-structured interview guide (S1 File) drawing on our previous research [12]. It was intended that emphasis be placed on matters important to each woman without the need to ensure that every woman answered all questions. Interviews were conducted (singly) by both authors who are female PhDs and academic psychologists with experience in qualitative research. JF is also a clinical psychologist. They were of the same generation as participants, with whom they had no pre-existing relationship.

We offered volunteers a choice of being interviewed by telephone or in person at the university campus. Interviews were audio-recorded, with permission. The interviewer also took detailed notes. For participants’ convenience and to accommodate telephone interviews, participants could give formal oral consent which was recorded at the beginning of the interview or return their signed consent form before the interview. Participants who chose an in-person interview could bring a signed form with them or sign a form before the interview began.

Before analysis, identifying details were disguised in or deleted from transcripts; a pseudonym was allocated to each woman. Both authors analysed interviews thematically, in accordance with well-established practice, using the steps described by Braun and Clarke [27], searching first for themes arising from the questions then for unexpected themes.

The interview transcripts are not available to other researchers. The Explanatory Statement given to potential participants included the following assurance: “Recordings and transcripts will be stored in a password-protected folder on a secure Monash University drive, accessible only to the named researchers.” The research was approved in every detail by the Monash University Human Research Ethics Committee, reference number 16700.

## Results

### Women

Substantial data and adequate diversity were achieved after 18 interviews (17 by phone, 1 in person) lasting an average of 45 minutes (29–83 minutes). One woman who initially volunteered to participate in the research withdrew because of her fear of discovery and retribution by her violent ex-partner. Signed copies of consent forms were returned by 7 women; 11 had their oral consent recorded. There were no non-participants present. Women’s birth years were distributed across the Baby Boomer range with only 6 of the 19 years unrepresented. Women were living in Victoria (9), New South Wales (3), Queensland (3), South Australia (2), and Western Australia (1) in a mix of rural and metropolitan areas. Ten women had a male partner; the rest had been divorced or widowed (5, including one woman whose female partner had died) or had always been single (3). Four women were born outside Australia, including in two Asian countries and a non-English-speaking European country. Most women (14) had a university degree (9 of the 14 had further qualifications), three women had a diploma or trade certificate, and one had completed secondary school. More than half of the women (10) were retired from employment; five were in paid employment, either full time (3) or part time (2); two had casual work; and one was a self-employed farmer. Most women also did voluntary work. Four women had experience in aged care, either professionally as a medical practitioner or pastoral care worker (Barbara, Gail, Joy) or as a volunteer (Rose). Although all but one of the women were recruited through the newsletter of a women’s health organisation (Jean Hailes Foundation)—the exception was a woman who was told about the research by a newsletter subscriber—the women revealed diverse experiences of life and ageing.

All participants were sent a brief report summarising the results. There were no adverse comments among the affirming responses.

### Themes

Mental health was recognised by all participants as significant. Four related themes (each with sub-themes) were identified in women’s accounts: Adverse influences on mental health, Contributors to good mental health, Other personal or social influences on mental health, and What older women need from others.

#### Adverse influences on mental health

Participants nominated Illness and disability, Financial insecurity, Maltreatment, and Loss and grief as the main contributors to poor mental health in older women.

*Illness and disability*. Almost all women identified an association between physical and mental health. Helen, for example, attributed her own good mental health to (among other things) having had very good physical health, and Kay said she realised that illness could “tip you” into poor mental health and was thus determined to remain well. A few women, such as Dorothy, described themselves, despite osteoarthritis, as having “relatively good health for my age” which contributed to their sense of wellbeing. Although Mei identified illness and disability as “critical,” she said that women need to accept them as part of ageing.

Having a mental illness could also precipitate further problems with mental health. Natalie, for example, said that managing her post-traumatic stress disorder (PTSD) was made more difficult at her workplace because “bosses are unsympathetic” and expect that staff “can handle any pressure thrown at you.” She was distressed by “the stigma attached to mental health problems, especially in the workplace.”

Two of the younger women (Ann, Quentin) reported long-term adverse effects of menopause on their mental health, and Ingrid reflected on how her early menopause continued to affect her physical and mental health at the age of 61. We are not characterising menopause as an illness or a disability, but acknowledge that women who experience untreated debilitating symptoms, especially when confronting other challenges, may become anxious and depressed as a result.

The most frequent link identified between illness or disability and poor mental health was that they limit activities that promote good mental health. As Fiona and Gail said, mobility contributes to independence. Ingrid’s untreated early menopause led to osteopenia, which prevented her from running. Withdrawal from this “loved” activity meant that she no longer participated in her athletics club, thus reducing her social links, to the detriment of her mental health. Similarly, Pamela spoke about the effects of her “debilitating arthritis” and chronic pain. She could no longer play golf, once not only her exercise but the focus of her social circle, nor could she travel the world meeting interesting people. Pamela was sad about these losses and said that the main threat to her mental health was lack of human interaction. Rose was acutely aware of the adverse effects of lost mobility and independence as a result of her work in an aged care facility, concluding that “physical limitations are depressing” even when accommodation and care are “excellent.”

Illness and disability were also identified as adversely affecting mental health because they disrupted a person’s sense of identity. Ellen had always been healthy and took no medication until statins were prescribed for high cholesterol. She was consulting a psychologist to deal with the distress of being “a person who takes medication” rather than someone who used “natural” methods to maintain good health. This sense of a disrupted self is applicable also to the women who described having to relinquish important activities because of poor health and to women who have lost their independence.

Women also considered reciprocal challenges to mental health for people close to them. Gail, for example, reflected on the stressful demands made by her frail mother. As a result, “I self-consciously don’t tell my daughters about my aches and pains.” Olivia said that becoming ill would have an adverse effect on her mental health, but so would illness or harm to her husband or children.

Two women, without prompting, said that they welcomed Victoria’s assisted dying legislation so that they need no longer stay alive when they were too ill to enjoy living. Mei wanted to be able to say “Enough! Goodbye,” and Helen said that “being able to decide when life is no longer satisfying is important to mental health.”

*Financial insecurity*. All women said or agreed that financial insecurity posed a threat to mental health, whether or not they had experienced it. Financially secure women (Ann, Ellen, Gail, Helen, Ingrid, Kay, Mei, Olivia, Pamela, Rose) identified insufficient superannuation and extended life expectancy as a major contributor to poor mental health. Pamela spoke of friends who could not afford to go to concerts and other expensive activities; this limitation on their social life adversely affected their mental health.

Dorothy had struggled financially after her husband left their 40-year marriage but worked to become “financially independent;” she was aware of older women who “live in poverty” and find it “damaging” to their mental health. Rose had “strong money management” and owned her own house without a mortgage, but “Bills, maintenance costs, they do worry you. … If anything happened to the car, to the house, to my health” it could be a problem, but “I try to suppress that thought.”

Women who were not financially secure or lived on a restricted income (Carol, Fiona, Joy, Louise, Natalie, Quentin) spoke of seeking food and entertainment they could afford. On retirement, Fiona had to modify her spending. Although not characterising herself as financially insecure (being able to maintain private health insurance), Fiona found it “hard to give up the things you love because you can’t afford it.” Barbara owned her home unit and lived on the aged pension. She also sought low- or no-cost activities and declared that “It’s important to be grateful for what one has.” As a nun, Joy accepted poverty as part of her life and lived “frugally” outside a convent. In her opinion, financial security is important but insecurity “doesn’t have to be a mental problem” for women, especially if “their kids prop them up,” although Joy added that she knew nothing about finances.

For a few women, financial insecurity was just one component of a difficult life that undermined mental health. Carol said that she had been diagnosed with bipolar disorder and had experienced both great success and bankruptcy. She lived in rented accommodation, but having little money was just one of the many threats to her health. Poor mental health appeared to have contributed to Louise’s financial insecurity by limiting her capacity to work. She lived on “next to nothing” and “used to worry about money” which threatened her mental health, but had been “reassured” by knowing that she could claim the aged pension in only four years. Natalie’s PTSD and her precarious financial state also had reciprocal effects. About six years before being interviewed, Natalie accepted a friend’s challenge and spent 18 months “homeless and unemployed” backpacking around Australia; she “loved” the experience and felt that it “freed” her. Nevertheless, Natalie could not afford not to work and said more than once: “I see my whole life as a homelessness prevention strategy.” This approach to life could be applied to other interviewed women.

*Maltreatment*. A few women had experienced significant maltreatment that had adversely affected their mental health. Maltreatment could be neglect or abuse by parents when they were children (Carol, Ellen, Natalie, Quentin), parental violence reappearing when they were adults (Ellen), intimate partner violence (Natalie, Quentin), financial abuse by a business partner (Carol), bullying or marginalisation by work colleagues (Fiona, Louise, Natalie), racism (Mei), and even the pervasive fear and violence of war and subsequent dislocation as a refugee (Quentin). We also note the woman who withdrew from participation because she feared discovery and retribution by her violent ex-partner.

Some participants knew women who had experienced intimate partner violence that had “profoundly adverse” (Ann) effects on their mental health. Barbara’s sister had an “abusive husband” and Barbara had encountered “elder abuse” through her work in an aged care facility; she “always heard older women being put down,” which is “bad for women’s mental health.”

*Loss and grief*. It was notable that all women described experiences of loss and grief as challenging to mental health. They spoke about their own losses and about loss and grief in general. In addition to the loss of pleasurable activities (and potentially the loss of self) resulting from illness and disability, they identified retirement from work, the death of or separation from family members, and the loss of country and community. Some women reported multiple losses.

When women retired, especially at a time not of their choosing, they lost meaningful work and social contact. Kay, for example, missed the “water cooler conversation” she enjoyed at work, adding that although she had “a wonderful dog,” he “doesn’t say, Good morning. How was your weekend?” Olivia found her lost social milieu at work “hard to duplicate” and Rose missed “meaningful” paid work.

Partners’ (in most cases, husbands’) retirement could also constitute a loss. Mei felt “irritated” by her husband’s constant presence; it cost her the comfort of routine and independence. Ingrid described her partner’s recent retirement as “a very challenging transition:”

from someone who … was not really at home very often, … and now suddenly he’s home 24 hours, 7 days a week. [We’re] trying to work out [my sense of] … ‘This is my house and this is my role! And now It’s the two of us all the time and you want to kind of do things that I do a different way.’ … That’s a transition that most people don’t really think about, or is joked about, … but I think it’s a significant issue for perhaps couples to address as they get older.

The death of parents could be a substantial loss with long-term effects on older women. Helen, who wryly described herself as “in the orphans’ club,” felt that she had recovered from her “intense grief” after her mother’s death in the previous year. Rose not only grieved for her mother (who had died nine years before) but for the calendar commitments and sense of being needed that visiting her mother had given her. Fiona’s father had died when she was 9 and she attributed to this great loss her failure to form lasting bonds: “I decided that it hurt too much when you love somebody, and that’s sort of put me anti-relationship.”

The death of a partner was reported as particularly painful. Pamela’s partner Penny had died two years previously; Pamela wept when she spoke of her, saying that grief still took her by surprise. Barbara’s husband had been dead for 19 years; she vividly described his illness and the effects of his death, characterising the losses that occur with age and the consequent “loneliness, living alone” as “like the leaves falling off.”

Separation and divorce also caused major—and often more complex—grief. When Joy had to leave her convent to live on her own (to work in another area), it was the major loss of her life which she described as “like a divorce,” although she thought that divorced people would resent her saying so. When Dorothy’s 40-year marriage ended (a few years before the interview) she “had to accept living alone,” saying that she, like “a lot of women,”

anticipate[d] getting older in the comfort of having someone who cares for you, but if … they care for somebody else, well, you have to care for yourself.

The loss of a child through death (as in Carol’s experience) or estrangement (Natalie) was acknowledged as particularly damaging to mental health. Natalie said that her ex-husband had turned her daughter against her and it was painful to be able to communicate with her only occasionally and in writing. Carol described in detail the death of her son at five weeks of age many years before; this seemed to be the focal loss of her life.

Quentin told of being born into a country experiencing civil unrest, going to school in an armoured van, and learning to use weapons from the age of 6. When she was 17, Quentin and her family came to Australia as “refugees.” She found “all the freedom” to be “abnormal,” “the lifestyle … completely foreign,” and the loss of her friends, her community, and a valued job so distressing that she contemplated suicide. Quentin was one of the women who continued to experience the adverse psychological effects of multiple losses. She reported a diagnosis of post-traumatic stress disorder, recalled a violent father and emotionally detached mother, had escaped and then (because of her suicidal son) returned to a violent partner, and experienced chronic pain after a severe injury. Despite these many problems, Quentin contributed actively as a volunteer in her community.

Carol, who wept throughout her interview, believed the death of her infant son to be punishment for a teenage abortion and told of a lifetime of loss: of people close to her, money, and prestige. Louise’s life story seemed to be one of loss of inclusion, of the isolation of being “different.” According to Louise, who was single and childless, “there’s no-one close in my life.” Although she had had “one or two” women friends, she had not found her “tribe,” describing herself as “not like the average female.” Louise reported diagnoses of depression and anxiety.

Although most of the women we interviewed had not lived such distressing lives, they all recognised the challenge presented to mental health of grief and loss. Barbara said,

In the last few years I’ve lost 3 siblings. … That’s the reality of life, you know; our roles have gone, our children have left us and they’ve got their own lives, and, you know, we’ve got deaths in our families, haven’t we? … For some people it’s less money, living on a pension, selling their family home. … Loneliness, living alone, women and homelessness. There’s just so many losses.

Joy was clear that poor mental health arises from failure to deal with grief and loss, and Barbara said, “we’re not very good in our society of letting people grieve.” Her experience working in an aged care facility led her to believe that unresolved grief could, as people aged, become a great burden, taking up “another load of space in their lives.”

#### Contributors to good mental health

Women were clear about what contributed to good mental health (apart from merely the opposite of influences on poor mental health): Social interdependence, Feeling valued (in which we include meaning or purpose), Physical activity, Good nutrition, and Having faith or belief.

*Social interdependence*. Interdependence of various kinds, from various sources, was identified as important to mental health. Women spoke of supporting and being supported by partners, children, other family members, friends, and the wider community.

Partners—usually husbands—were said (either directly or implicitly) to be particularly important to mental health. Good relationships provided reciprocal support. Ellen described her husband as “invaluable” and her “best friend,” Olivia said she had “a terrific spouse,” Ann had “a happy marriage” with “a good husband,” and Natalie’s “wonderful” husband was, she said, “the saviour of my life” and a vital component of her “support system.” Pamela grieved the loss of her partner, Penny, recalling how they “supported each other.” Another widow, Kay, said that being alone made it difficult to stop worrying and that she missed her husband’s “calmness and reassurance.” She emphasised that it was not the case that “loneliness or aloneness means necessarily going on to have a mental health problem,” but thought she had to be active in guarding against the possibility. Dorothy, who was divorced, had to “work hard to make sense of living alone” after assuming that she would have “someone to grow old with.” Kay was considering leaving her country property as she aged, and said, “If I did have a companion in my life, male or female, who could live with me, I could live here longer.” Companionship, not (or not just) a sexual relationship, appeared to underlie the importance of partners to mental health.

Children were mentioned less often but their impact on women’s mental health could also be profound, although different from partners’ influence. Natalie’s son was part of her mutual “support system” as were Olivia’s children, but Olivia commented that, because “my children turned out well,” they did not cause her to worry, adding “it’s bad for mental health if children do.” Carol said that she was “anxious all the time” about her children. Ellen was pleased that the last of her three children had recently left home; she found it to be “a good thing” to have “children independent,” allowing her and her husband to “enjoy their freedom.” At the same time, Ellen made an effort to ensure that her children were “not made to feel that I depend on them.” Similarly, Gail does not “tell my daughters about my aches and pains,” wanting to maintain good relationships and not to be dependent on them, and Rose, having been responsible for her mother’s home care, said, “I don’t want to impose on my daughter,” preferring to move to a nursing home when her needs increase. The care some women took not to “burden” their children reflects the mental health implications of close relationships.

A few women talked about being without children, Carol because hers had left home and she found “the empty nest” to be a lonely experience that adversely affected her mental health. Fiona, who “like Julia Gillard [past Australian Prime Minister] … wasn’t deliberately barren,” had come at the age of 50 to feel “a bit left out” and resentful as other women talked about the new life stages of children leaving home and grandchildren. In contrast, Dorothy described herself and her husband as “childfree:” “Fortunately we’re in that age group when we had access to contraception and didn’t have to succumb to pregnancy if we didn’t wish to.”

Gail, Rose, and Mei told of the challenges of looking after very old (approaching 100 years) parents and parents-in-law and the accompanying conflicting emotions. Rose’s mother had died nine years earlier after years of care from Rose; she was both saddened and thankful to have been relieved of the burden. Gail enjoyed frequently caring for her grandchildren, saying that it “made her feel better” after dealing with her mother. Mei said that her husband and his mother both appreciated her care of them but that she was now in what she described as the third phase of her life and was beginning to withdraw from these burdensome responsibilities. According to Mei, the first phase is preparation, during which you do your best not to disappoint your parents; the second phase is looking after others through family and work; and the third phase is looking after yourself.

Friends were said to make particularly valuable contributions to older women’s mental health, through reciprocal support, companionship, and the sense of being known. Kay thought it was important to “create a bunch of friends you can rely on” and said there were “three or so … long-term friends … who I feel at the drop of a hat I could ask them something,” although she tried to be “independent.” Helen delighted in her “inclusive group of friends,” Mei said that it was “important to spend time with like-minded people,” and Ann found her relationships with female friends to be particularly helpful in avoiding loneliness. Ingrid and Fiona both said it was important to meet with “young” or “younger” people, which Ingrid said was “really energising.” Rose stated unequivocally that meeting others “is important to mental health.” Quentin’s experiences of abuse and neglect led her to conclude that “friends are more helpful than family;” she provided occasions on which she and her friends can be safe and “support each other” in managing their similar problems.

There were different views on the extent to which older women should confide in friends, with Fiona (like Quentin) urging others to “keep your friends and talk about things; if something’s worrying you, talk about it.” She described it as “like an accidental counselling,” adding that “often your life experiences can help them.” In contrast, although Gail saw friends as contributing to good mental health, she said, “I prefer to pay a counsellor than to burden others” with any personal problems.

The wider community was also identified as an essential contributor to good mental health through social support and the opportunity to contribute to community life. Olivia identified “social contact” as an important contributor to mental health and said that she pursued interests that fulfil her social need, including a book group and University of the Third Age. Her advice to older women was, “Do what you can not to be isolated.” Dorothy described her rural community as “an essential component of my mental health;” she felt known and cared for: “You have some identity in a place like this, and I think that helps with not feeling lost or alone.” Ingrid said,

As you get older, it’s particularly important to have things that will stimulate you and interest you and hopefully form some kind of connection with other people, whether you see that through the church, or in your community, or mutual interests.

Community engagement often included volunteering. Quentin thought it was “important to participate in the community” for “the sake of your mental health,” explaining that “Giving to other people keeps me sane.” Dorothy said participation in community groups not only gave her “dates to put on your calendar” but that “being part of a community is also part of being purposeful.” Through volunteering in her rural community, Dorothy had “created a bigger network of people who know where I am, what I’m doing, who care for me, and vice versa.” Fiona said, “I like feeling that I’m contributing to society and the volunteer work is important to me. … It’s better than work because I’m doing the things I want to do when I want to do it.” She added, “I don’t have children, [but where I volunteer] … I’m mixing with those age groups. It’s maybe not quite the same but I’m finding that satisfying.”

*Feeling valued*. Women’s accounts of what made them feel valued were informative, especially in their illumination of how experience influences mental health. Feeling that one had made a meaningful contribution was particularly important; this could come from helping others and volunteering, participating in or supporting research and donating to artistic endeavours (Pamela), being a blood donor (Dorothy), or working in a caring role or a job that was valued. This last was problematic. According to Louise, when she worked “in care” it was “the most rewarding job and the most low paid.” Pamela said that, although she had felt valued through her profession, she disliked that it was her role and not herself that was valued, and Ingrid said she felt unvalued as soon as people learnt that she had retired, adding that “Australian society” was “very influenced by what your perceived value is according to your profession.”

Being appreciated also made women feel valued, whether it was love and attention from a partner, being sought out by grandchildren or friends, or being paid attention and listened to by community members or health professionals. Rose said that judging herself to be responsible made her feel valued rather than being valued by others. Mei was alone in saying that nothing made her feel valued, although there appeared to be a cultural or personal reluctance to assess herself as valuable as well as a desire to convey that she contributes and helps others without expecting any reward.

*Physical activity*. Most women said that physical activity contributes to good mental health. The activities they described spanned gardening, walking, swimming, group classes, bushwalking, dancing, running, cycling, horseback riding, and skiing. Physical activity was identified as part of taking responsibility for one’s health, a source of pleasure, a contributor to social contact, and a means of overcoming challenging life experiences. Women also spoke of the problems they encountered with their sense of wellbeing when physical activity is reduced or denied.

A sense of personal responsibility could contribute to personal wellbeing. Dorothy, for example, said that “Taking some responsibility for your health is important; … physical activity is part of that taking responsibility.” She recalled her surgeon saying, after surgery on her back, “Stay lean and stay active,” and Dorothy had committed to doing just that.

Women made it clear that their diverse activities could be sources of great pleasure and that physical activity must be pleasurable if it is to be maintained. Activities could be practices developed over decades, although some women had become physically active only recently. Mei said that she had been “brought up to be a lady” and that exercise was unladylike, but now thought that older people need both physical and mental activity and had come to enjoy participating in gym classes. Olivia said she was “never a sporty person” but now cycles three times weekly and does personal training for strength, flexibility, and bone health. Helen attributed her good mental health to her good physical health, but said:

It’s no use saying, ‘Oh’, you know, ‘You’ve got to keep fit, you’ve got to go to a gym’. I hate gyms! But I do enough in the garden, I bushwalk, and … I’ve gone back to yoga. But it’s no use keeping fit if you’re doing something that you don’t like.

The most notable feature of physical activity reported by women was its important role in social contact. Fiona volunteered to weed bushland and participated in bushwalking, in each case as part of a social group that she valued. Dorothy said, “Part of my social contact … is getting out riding with my friends. Most of my riding friends are women … who are 60-plus.” Kay saw a direct link among physical activity, social interaction, and good mental health, saying:

I go to a very—you wouldn’t call it strenuous—an exercise class, and a lot of the ladies are late seventies, some of them are in their eighties, and I’m not sure that they have any other social contact. So for them it’s the highlight for the week.

Physical activity was also identified as a means of managing distress and mental illness. Barbara said she endured her husband’s illness and death by walking and now maintained her exercise program. Rose said that, when she is sad, “I pick myself up and walk.” She also did group yoga because it was “calming, relaxing” and to “keep myself busy,” adding that “meeting others is important to mental health.” Quentin exercised with a friend “who has psychological problems” and who benefited from “the fresh air,” activity, and company. Louise used exercise specifically to manage anxiety and chronic fatigue. In her forties, Ellen, too, had had chronic fatigue and contemplated suicide; she challenged these problems with as much exercise as she could manage. Natalie, who had lifetime challenges to her mental health and had been diagnosed with PTSD, swam daily. When asked whether physical health played a role in mental health, Natalie replied “150 percent yes.”

It can be difficult for women’s wellbeing if physical activity is constrained by injury or illness. Kay “used to be able to garden for hours on end, but now that’s just not possible any more.” That made her sad, although she hoped to find substitute activities. Ingrid was “very fit and running half marathons” until she had a stress fracture and was diagnosed with osteopenia. She described her trajectory as:

from someone who was very fit and running between 20 and 30 kilometres a week. And I belong to a masters’ athletics club which was a big social part of my life, and I was told … walk carefully because you’ll probably have a major fracture to your neck femur within the next five years. … It has really upset met and had enormous consequences.

Nevertheless, even women whose previous activities had been curtailed said they tried to find (often sociable) ways of being active and that this was part of maintaining good mental as well as physical health.

Ingrid pointed out that it is not just injury or illness that can prevent physical activity; there are socially gendered obstacles:

I think that activity, whether it’s walking or running or cycling or swimming or whatever it is, but regularly, is particularly important as we age, and perhaps something that women have often neglected for family reasons. So the guys might go out and play organised sport and the women would be home doing something else. Organising the house or shopping or looking after the kids, that kind of thing.

Pamela had concerns about the way exercise is promoted to women that may not assist their physical or mental health. She wanted “to support older people in things that aren’t sport,” adding that she “would say ‘exercise’ but it has bad connotations” of “trying to look like Jane Fonda used to look.” According to Pamela, a women’s health website that she valued needs “images of older women exercising but not all looking fit and fabulous” and better representation of body types, to make sociable physical activities seem accessible to all older women.

*Good nutrition*. The majority of women said or agreed that good nutrition was important to older women although the role they attributed to it in maintaining good mental health varied. A few of the women who identified a direct connection linked “good eating” (Barbara) with being active and “engaging my mind” (Fiona) as underpinning good mental health. Ingrid distinguished “attention to a healthy diet” from “dieting” as beneficial to mental health. Joy said that nutrition and physical activity are “vital,” linking them to “neural plasticity.” Helen spoke of the value of having “a nice vegetable garden” because

I’d rather go and pick some lettuce and some broad beans fresh out of the garden than buy, you know, broad beans that could be two weeks old. Nutrition plays a huge part.

Dorothy had another understanding of how nutrition and mental health are linked. “Doing something good for your body” makes you feel “positive,” but not eating well leaves you “feeling guilty about doing something you know is not good for you.” Only Pamela asserted that good nutrition is not relevant to mental health “unless you’re concerned about how to pay for it or can’t afford it.”

*Having faith or belief*. A few women invoked the role of faith or belief in contributing to good mental health. They referred to generic spirituality, Eastern practice, and Christianity. Ellen practised the Ayurvedic tradition, which she described as based on the belief that health and wellness depend on a delicate balance of mind, body, and spirit, an association echoed by others, including Barbara: “We are body, mind, spirit, and spirit is needed in tough times.” Although Helen was “not religious at all,” she thought that “sometimes having that faith can actually help you get over grief.” Joy said that she had been helped in coping with life’s vicissitudes through her Christian understanding that “suffering is part of life” and the need for “accepting and surrendering.” In her view, “God and spirituality are important to a positive life,” although it is not essential to be Christian. Mei also said that one’s philosophy of life is influential, specifying that “the Chinese approach to happiness is contentment and acceptance; don’t fight what you can’t change but learn to live with it.” After her interview, Rose sent an email in which she wrote,

There’s one more aspect I like to add, and that’s a ‘faith’. I am a catholic and I found that it has helped me. Over recent years I found in particular prayers and some reading and attending teachings and reflections, in groups or not, calming and contentment.

#### Other personal and social Influences on mental health

Women’s accounts supplied other influences on mental health, whether associated with the person (personality, intimate relationships and sex) or with society (constructs of ageing, gender, culture).

*Personality or character*. According to Rose, “Mental health … is to do with character.” She related this generalisation to herself as having “a tendency to be sad,” advising older women to “avoid negative people.” Mei stated this approach more colloquially as “personality or a familiar way of thinking of a glass half full or half empty” while confronting the knowledge that “the whole world is going down the gurgler.” Dorothy said that people with good mental health “focus on the positive” and added that her capacity to think positively was “a consequence of the person I am.” Similarly, Olivia described herself as “resilient” and therefore able to recover from difficult life events, and Barbara attributed her own good mental health to “discipline in my thinking:” not dwelling on life’s problems and choosing what to worry about. Ingrid said that mental health in ageing depends on “what sort of personality you are,” narrowing it down to “whether you’re an introvert or extrovert.” Joy was concerned about older women seeing themselves as victims, saying that “those who feel like victims are likely to have had this attitude all their lives.”

Although these views could be seen as making a circular argument—to have good mental health in older age women need to have good mental health—it can also be understood as guiding women to reflect on and re-evaluate their sense of themselves in the world. Joy said explicitly that older women need “education and help … to reframe adversity” and “to challenge a negative outlook.” If disability comes with ageing, Joy added, women need to find a new understanding of “what they and their bodies can do” and learn “to accommodate change as best they can.” Gail said that “Preparation for healthy old age must begin young.” However, as Pamela said, “We weren’t taught to be parents and we’re not taught how to be old.”

*Intimate relationships and sex*. Intimate relationships were acknowledged as having a profound influence on older women’s mental health. Helen, for example, said:

I have an extremely loving partner. I mean, life isn’t a bed of wine and roses, but I’ve had the same consistent partner … for 50 years. And that is a great precursor to good mental health.

And Rose, who had separated from her husband more than a decade before, thought that having a partner “might help my mental health.”

Most women described beneficial or at least benign partnerships and their contribution to good mental health. Where partners were abusive (physically, emotionally, or in other ways) they could have the opposite effect. Quentin was living with a violent partner so that she could care for their son. She felt protected to some extent by a restraining order, but described the main threat to her mental health as her husband’s dislike of her work in the community, saying, “I feel a bit like a bird in a cage.”

Although almost all participants mentioned male partners, Pamela, the participant whose partner was female, gave an account of her life that was consistent with those of other women. She seemed surprised to be asked if she and Penny had encountered any problematic attitudes arising from their same-sex relationship, saying that they had thought it would be an advantage if they had to go to a nursing home because they could be in adjacent rooms, whereas female-male couples were likely to be sex-segregated.

Women who had lost a partner (through death or separation) and women who had always been single could find it difficult to develop intimate relationships, if that is what they wanted. Dorothy, for example, had tried “internet dating” but had concluded that “it was mostly people scamming older women.”

Sexual relationships and activities, while not discussed by most women, were evidently potentially influential on women’s mental health as they aged. Ingrid spoke of a close friend who had sought medical help because she was finding sexual intercourse difficult. Several doctors were reported as telling her, “Well, that’s to be expected.” When older women want to be sexually active, the experience can be, as Ingrid said, “so ironic! At this age, where you finally don’t have to worry about getting pregnant any more, suddenly you can’t have sex: It’s so uncomfortable.” Ingrid said that sexual health “should be automatically part of the consult. … It’s not just ‘Slap on a bit of Vaseline and go for it’.”

Dorothy warned that support for older women who wanted to initiate or maintain sexual activity should not be generalised into expecting all older women to be sexually active. She was “dismayed” when a prominent website for women had begun to include articles that gave that impression; Dorothy found them “offensive:”

because it assumed that either everyone was in a relationship with another woman or another man, or that they were masturbating. And I thought, this is really putting pressure on people. … It is a negative pressure that, ‘You older women would be happier if you had a sex life.’

Dorothy thought that sex “would be nice in relationship, … but I wish there weren’t that pressure.” Her experiences of sex with men since her divorce had revealed that “men have impotence problems and you have dryness problems.” She concluded that “Sex is complicated.”

*Ageism*. Some women spoke about social constructs of ageing and attitudes to older women and how they affect mental health. Olivia said that it is “too easy for society to discount older people.” According to Natalie, “women from about 60 onwards are a very vulnerable group” because of inadequate financial support and the risk of social isolation; this is compounded by a feeling of being “invisible” (Ann). A sense of no longer being recognised as a valuable member of society was identified as restricting employment opportunities, which Carol linked directly to “ageism.” Dorothy said that she did not describe herself as “retired” because of the connotations of ageing:

I like to consider myself as self-employed. I choose what I do each day. But look, the ‘retired’ word has a stigma attached to it, … just as the word ‘pensioner’ does. I try not to use it. On my application for various things I put ‘farmer,’ because ‘farmer’ describes my lifestyle.

Ingrid also asserted that society does not value older women when they are “not working” and subjects them to “condescending and patriarchal attitudes.” Assumptions are made about older women, she said, without understanding who they are or the lives they have lived. Barbara included the medical profession in this generalisation, saying that “doctors don’t care about” older women “because they can’t treat them.”

Concern about poor management of older women’s mental health, arising from ageism, was what Ingrid said had motivated her to volunteer for the research. She thought that older women’s health concerns are dismissed as being “all in their heads,” normalised as an inevitable component of ageing and, as a result, “neither investigated or assisted.” As an example, Ingrid said that sexual problems in older women, including vaginal dryness and low libido, are given inadequate attention, adding that “good health is not just for young people.”

Pamela found ageist attitudes confronting and wondered how they could be improved, suggesting that “good news stories” about older women might contribute. Similarly, Natalie spoke of the necessity of “a positive profile for the needs of that group as it gets bigger, and it will,” proposing that “Kerryn Phelps [a senior doctor, at that time a politician] or someone like her … would make a brilliant public face for older women’s mental health.”

*Gendered differences in ageing*. Some women reflected on what they identified as gendered differences in the experience of ageing that could affect mental health. Barbara noted one positive difference: that women “are more resilient than men” because they are socially encouraged to share their emotions. She reported that men in the aged care facility where she works told her they envied women because men could “not talk meaningfully” with other men, having been trained only to “discuss work and sport.” Barbara hoped that the Baby Boomer generation was changing this gendered difference.

The other gendered differences were to women’s detriment, and extended beyond the likelihood of being far less financially protected than men, although women also commented on this. Ingrid, for example, said of Baby Boomers:

Women’s roles are still, I think, very predominantly traditional. … Many women are still caring for their family and having that nurturing and a lot of home duties, even if they work full time and have a career; I think that’s still a significant part of their roles. … In the sixties-plus, certainly, I observe that a lot of my friends and relatives are now starting to confront quite significant health issues as well as helping to negotiate the care of elderly parents.

Gail appeared to endorse this perspective when she said that her retirement (in a few years) would not be a problem for her because “my identity is not tied to my profession” and “I’ve always thought being mother and wife is more important.”

Traditional gendered roles are also evident in assertions that women find it difficult to claim care for their own needs when they feel responsible for others’ needs. Natalie said that women “mother the world” and “it’s a necessity” to “claim the right to self-care.” Joy had found that some women she knew “feel selfish” for even considering themselves, with adverse effects on their physical and mental health.

Because women tend to outlive men, Joy thought that widowhood was particularly problematic for women who had occupied traditional gender roles. Their caring identity (as wife, mother, employee) has been lost and they must “search for a new identity.” Joy had found that some older women “are scared of being alone” and they become anxious. In contrast, women who had always been single and childless (such as Fiona) can feel that they experience the adverse effects on mental health of failing to fit the traditional narrative of women’s lives.

Ingrid expanded on her comments about ageist attitudes that affect women’s health to include differences based on gender:

A lot of issues that women have presented with [are treated] as all in their head, or not given the same significance as if a male presented with an issue of perhaps similar significance to their lifestyle. … Recently [my partner] went to his GP with having difficulties with an enlarged prostate, and I was just astonished the amount of blood tests and investigations and referrals that he was offered, as compared to when I go to the doctor and they, perhaps being an older person and suffering with things like low libido and dryness of vagina, it’s just more or less, ‘Oh, well, that’s something that happens to older ladies’. We go, ‘Oh, really?’

Ingrid noted this “even for female doctors.”

*Cultural differences*. There were indications of cultural differences that influenced the experience of ageing, with implications for mental health, although this was a minor theme in this set of women. Mei reflected on the “ingrained” Confucian influence on her life and how it was at odds with Western thinking. She was brought up to think of country first, then community, then family, then self. In contrast, she described Western thinking as “very individualistic.” Mei had become more aware of the Asian cultural influence with age as her (Western) friends began “fighting over their parents’ wills with their siblings.” Mei thought this was “inappropriate” because children should “respect what their parents wanted.” However, Mei also said that living in Australia had made her “more assertive” as she aged, and she had benefited from this cultural difference.

Invoking another kind of cultural difference, Quentin described arriving in Australia as a refugee from a war-torn country and finding the culture shock and loss of (even destructive) familiar things to be extremely damaging. It was evident that cultural differences, as well as trauma, continued to play a role in Quentin’s life as she aged, with the significance of events from her childhood not fully understood until maturity, motherhood, or more recently.

#### What older women need from others

Women specified what they needed from others in order to improve their mental health as they aged. They spoke about public education, ensuring that older women still had roles in society, provision of adequate services and resources, and what they needed from healthcare providers. It could all be summarised as treating older women with respect and ensuring that their lives had meaning.

When women described what gave them pleasure and what might help them and others to maintain good mental health, details were diverse, including volunteering, “socialising,” “talking with younger people,” spending time with grandchildren, travel and the associated planning and memories, dancing, singing, drinking coffee, bushwalking, gardening, yoga, swimming, “keeping fit,” reading, seeing opera and ballet on film, and attending concerts, theatres, and cinemas. Fiona urged older women to “follow your dreams” and keep company with “happy people” while avoiding “sad movies,” emphasising that they should “try to find the fun in … life.”

The thread running through these diverse details is summarised by Joy, who said that the primary contributor to good mental health and source of pleasure, including in older age, is “having a purpose in life” and “being able to reach it.” Much of what they wanted others to contribute to the lives of older women can be seen through this lens. The contributions they needed were both general (such as changing attitudes to ageing) and personal.

Gail, Joy, and Pamela were among those who said that governments and other authorities should educate the community as a whole about how to grow old in good health and to reframe ageing as not a problem but a strength. One component of this is not assuming that all older women are the same. Fiona, for example, said,

I’m a single female. I’m not homosexual. I’ve never married. And I just feel sometimes that my statistics don’t appear as often as others, and particularly politicians always talk about mum-and-dad voters. And I think sometimes single females can be a bit invisible.

Kay wanted to regain “this sense of community that we’re seeming to lose” in which “people look after each other, or look out for each other, and, if something is wrong, they would do something.” Other women had suggestions for strengthening older women’s engagement in community life and their capacity to contribute. In particular, Olivia wanted ways to be found for older women to use their skills and experience in mentoring younger women, both in and out of paid workplaces. She also called for more “proactive” groups “along lines of the senior citizens’ centres that used to be all over the suburbs,” which could provide “interesting activities.” Joy described these as centres that offer “socialisation, exercise, and the opportunity to contribute. … Not just knitting and sewing.” If women must enter an aged care facility, it was recommended that facilities should provide social and intellectual stimulation and they should not be isolated. Barbara said there should be activities for people of all ages on the same premises, with child care given as one example.

Participants advocated strongly for the provision of adequate, diverse services for older women. Suggested services included improved access to organisations such as the University of the Third Age, dedicated community buses or other forms of transport to deliver people to group activities, support “to be able to stay in your own home with the amount of help that you need” (Helen), and, where necessary, “subsidised sessions with a psychiatrist or psychologist” (Ann). Fiona, who said that “enjoying nature is good for my mental health,” urged authorities to maintain parks and reserves as a contribution to community wellbeing. Women’s support groups were deemed important and worthy of government funding. Louise said that she had belonged to groups for anxiety and chronic fatigue, finding them valuable but always “worried about money.” Natalie wanted governments to “invest in us financially.” Pamela spoke for other women when she asked, “Who do I lobby to get services for older women?”

Participants said that older women should receive accurate information about “their rights” as well as “what sort of resources there are, particularly for their needs” (Ingrid). Information could come not only from their GPs but also through social media, especially Facebook. Helen said that, whether information was distributed in print or online, it would be “very helpful to women,” especially in subscription format, by which she meant “digestible pieces of relevant information sent out regularly.” Should women need to contact services such as Centrelink (social security payment organisation), Fiona said that the waiting time was “not acceptable” and the government needs “to employ more staff to help people.”

Among possible resources, there were diverse views on the role of technology. Olivia called for “government and organisations not to encourage things that keep people at home, such as IT solutions.” In contrast, Ingrid wanted older women to be helped to be technologically competent, saying that “some older women” can be “very intimidated by technology” which is “isolating and very difficult” now that “everything is online.”

I think that getting them the confidence and the right people to actually teach them and to say that, ‘You can do this!’ would be … useful. Because often the men have used technology perhaps a bit more than women, and I think, for a number of older women, even trying to use things like mobile phones. … Connecting with other people via Facebook could be … very useful for older women.

Women identified a good GP as central to mental health. Somewhat cynically, Ingrid said that it is “really important to try and get a good health professional that will work with you and listen to you. And good luck with that.” According to Natalie, who had found a supportive GP, “Ninety-nine percent of GPs don’t listen to me. … Health professionals usually tell you to suck it up.” About half of the women said, without prompting, that health professionals should take time to listen to women rather than act on assumptions about what older women need. Joy said, “GPs should stop medicating older women so much. Listen to them and find out what’s causing the sadness and anxiety.” In parallel with this concern about investigating the psychological underpinnings of physical ailments was a concern not to dismiss physical symptoms, with Ingrid saying, “It’s important for both the woman and the health professional to acknowledge it’s not just in your head.” Quentin wanted medical specialists, in particular, to be aware that their patients’ lives can be complicated, and not to deal only with the matter that took them to the consultation. She thought “Women need to know how to train professionals in recognising a complicated life.”

It was common for women to want people, especially doctors, to accept that the old woman in front of them was (as Helen said) “once young, vital, and capable” and to “look past the persona that you face” who might be frail and diminished. This approach was implicated in treating women with respect. When asked to give advice to those who care for older women, Dorothy said:

Respect them as an individual. They’ve had a life. They’ve got a story. They were once young and beautiful in a wedding photo, or achieved something. … Treat them as you’d want to be treated.

Helen was one of those who added “dignity” to respect, and Natalie’s succinct advice was “Take us seriously.”

The two women who were glad of assisted dying legislation in states where it had been enacted wanted it to be more widely available. Helen said that she would rather die than “go into a nursing home.” Mei said that she used government websites for making a living will and would do so for power of attorney; she recommended that other women took advantage of what state and federal governments made available.

## Discussion and conclusion

Interviews about mental health with Baby Boomer women in Australia yielded evidence of diverse influences on their mental health and what others (such as policy-makers and health practitioners) can do to contribute to optimum mental health in older age. These insights into personal understanding of mental health and wellbeing add depth and nuance to what is already known. This is the first published research of this kind conducted in Australia; it also contributes to knowledge in countries with similar cultures. We have not reduced mental health to a single binary construct of being sick or well but have shown how experiences and their meaning have a lifetime influence on mental health in older age. Our approach fulfils a long-argued need to focus on psychological wellbeing, rather than psychopathology, in adult life [28]. The consistency identified between these results and the literature adds validity to our conclusions.

Contributors to good mental health were identified as social interdependence, feeling valued, physical activity, good nutrition, and having faith or belief. The importance of social interdependence for the wellbeing of older women has been found by others in Australia [29] and Canada [30]. Social engagement is linked to feeling valued, another important contributor to mental health, as was found in a UK survey of people aged 50 and older [13]. Purpose in life (what we have described as a meaningful life) was found in a meta-analysis to be associated with social integration and wellbeing [31]. Physical activity is now known to improve mental as well as physical health. For example, it was found in Germany that wellbeing in older age is associated with maintaining or increasing healthy physical activity, even when the older person has concurrent problems with physical health [32]. Physical activity is also, in itself, a source of pleasure [33]. Being active may involve the enjoyment of being in the natural environment, as our participants reported, which is associated with reduced cognitive decline in people in their seventies [34]. There were diverging views from our participants on the contribution of good nutrition to mental health, reflecting changing scientific opinion. While the effect of declining cognitive function on nutrition has long been a feature of ageing research, the role played by good nutrition in mental health is increasingly being acknowledged [35]. There is other evidence that faith is important to the mental health of older people because it provides engagement and social support and encourages health behaviours [36]. As we also found, benefits are claimed not just for the conventionally religious but also from spiritual practices such as meditation, expressions of gratitude, and enjoying the world of nature [37].

The adverse influences women identified—illness and disability, financial insecurity, maltreatment, and loss and grief—are consistent with other research. The contribution of good health and mobility to the wellbeing and mental health of older women in Australia is clear [10, 12]. The detrimental effects of financial insecurity are also established [4, 10, 28]. Elder abuse and its harmful effects are well known (for example, [38, 39]). Our own analysis of data contributed by the 1921–26 cohort of the Australian Longitudinal Study of Women’s Health (Waves 1–6) found that poor mental health is associated with adverse life events and experiences of interpersonal violence [12]. Our interviews with Baby Boomer women contribute insights into the experiences of maltreatment over a lifetime, not just in older age. Similarly, the influence of accumulated loss and grief arises not only from the deaths of significant family and friends because of age but, as these women revealed, losses throughout life that are not only of people but also of recognition and esteem, physical fitness, familiar environments, and other components of a fulfilling life.

Other influences on mental health were associated with the person and society. Personality, expressed as attitudes to ageing and life in general, has also been identified by others as contributing to good mental health. For example, women with osteoarthritis, interviewed because of their participation in the Longitudinal Study of Women’s Health, managed pain and functional impairment by conscious application of stoicism and daily psychological adjustment to the losses and demands of disability [40]. Our participants stressed the value of companionship with someone who was not necessarily a sexual partner. Nevertheless, as they aged, women wanted to be seen as potentially sexual beings, as others have found [21], while at the same time not being expected to be sexually active if they did not want to be. There is a social construct of sexual intimacy as heteronormative [21], with penetrative sex privileged [41], which can be challenging for non-heterosexual women and those without a male partner. It is important to note that analysis of data from the Australian Longitudinal Study on Women’s Health found that women in their seventies who remain single and childfree do not constitute a ‘problem’ group of lonely adults but tend to have higher education and to be more financially secure than other women [42]. Nevertheless, our participants (whether partnered or not) experienced the adverse effects of the social construct of ageing as debility, decline, and a burden on the young, as found elsewhere [1–4, 10]. Our participants echoed the finding that that older people themselves construct ageing as a gendered phenomenon [8]. Women see themselves and are seen as more resilient and emotionally mature than men, consistent with survey results [10], but they were also less financially secure than men and were aware of the social expectation that women should look after men and much older family members. Our results were suggestive of cultural differences in attitudes to and experiences of ageing that could influence mental health, consistent with a review of research published in English and Chinese characters [7]. As indicated by our participants whose origins were Asian, Chinese studies placed more of an emphasis on life satisfaction and family that Western studies [7].

It is evident from our results that experience and circumstances throughout life—proximal and distal—could have profound influences on women’s mental health in older age. This conclusion is consistent with evidence from data on more than 17,000 respondents to a European survey on ageing and health, in which it was found that, although health in older age (mean ~ 70 years) was associated with socioeconomic status in childhood, it was “almost fully mediated” by education and socioeconomic status in midlife [43]. While acknowledging the long-term influence of conditions in childhood, these researchers argued that the effect is indirect and incremental, and that interventions to improve socioeconomic influences on health in old age should continue throughout adulthood rather than be confined to childhood [43]. Similarly, a pathway analysis of Australian Baby Boomers found that, although adverse childhood events were indirectly associated with poorer quality of life and life satisfaction, more proximal adult events had greater influence on people in their early sixties [44]. Similar results were achieved by a large US survey of people aged 50–74 years [45].

The lifelong impact of circumstances and experience on older women’s mental health means that social structures that support and enhance the lives of girls and women at all ages will benefit older women and increase the potential for their continuing contribution to society. However, some requirements are especially important to older women. They wanted public education to counter ageist stereotypes, purposeful roles in society for older women, adequate services and resources, and respect from healthcare providers. A few years ago, an Australian politician published a book about ageing [46]. He emphasised the role played by Baby Boomers in reshaping the constructs and politics of ageing and argued for recognising, supporting, and promoting the continuing contribution of older people to the community [46]. Our participants appear to think that this mission is yet to be accomplished. Their assessment is consistent with results from Canada, where service providers were found to recognise the necessity of social participation for ageing Baby Boomers, but revealed that their organisations tended not to have specific policies in place to promote social participation [30]. The researchers drew on the WHO’s Checklist of Age-Friendly Cities [47] to raise questions about what could be done by organisations and providers to improve affordability, inclusion, accessibility, range, and fostering community integration [30]. The researchers concluded that there was not only a need for increased funding but that Baby Boomers themselves need to be consulted about what they need and want [30]. Evidence from an analysis of sickness fund agencies in Belgium revealed the expectation that people will age well by managing their own health, thus (perversely) denying agency for older people [48].

Healthcare providers were urged by our participants to listen to older women and treat them with respect, which would seem to be a fundamental component of a civilised society. It is notable that the women who spoke to us thought that older people, especially older women, were not always treated this way. The implications of these results for those who provide health care to older women is that each person should be treated as an individual with a history. Rather than focusing on symptoms, providers could attend to enhancing whatever means there are of ensuring that older women experience social interdependence, can enjoy appropriate physical activity and good nutrition, and that their lives include purpose and meaning. This approach is likely to contribute to good mental health.

These results suggest that policy-makers should ensure that, at the most basic level, policies and public statements by those in authority do not reinforce ageist stereotypes. More positive action would be to introduce policies that reflect the contributions of older people to society and encourage even greater participation, as a means to promoting good mental health. To the same end, service delivery should be based on evidence such as ours and consultation with those who use and are likely to use the service to ensure that it is non-discriminatory and conducive to good mental health.

Our results have implications for the mental health of older women and society as a whole. If older women experience a state of wellbeing in which they are able to use their skills and capacities, feel valued, enjoy gratifying relationships, and are enabled to continue contributing to communities and society, this not only improves their quality of life but benefits their communities.

## Supporting information

S1 File Interview guide. (PDF)
