# Supplementary material for: Promoting older women’s mental health: Insights from Baby Boomers
Source: PLoS One. 2021 Jan 12;16(1):e0245186. doi: 10.1371/journal.pone.0245186 (PMC7802969; doi:10.1371/journal.pone.0245186)
Supplement: S1 File — (PDF) [file pone.0245186.s001.pdf]

Preamble

[*If by telephone*: Is it still convenient to talk now?]

[Establish that she has read the explanatory statement]

As you know, this research is about older women's mental health and wellbeing. Do you have any questions about the research before we begin?

May I record our conversation to ensure that we accurately capture all that you say?

If she has not sent a signed consent form: Do you consent to participating in this research project?

Reason for volunteering

I'm interested to know why you volunteered for the research. Is there something you particularly wanted to say, or a story you'd like to tell?

[Adapt to sequence in what follows to suit what she has said already]

Comment on research results

In the document we sent you about the research, we said we'd like you to comment on research that we recently completed. We had data from surveys of more than 12,000 women, born from 1921 to 1926. We found that most women maintained good mental health from their early seventies to their mid-eighties. A minority, however, either experienced generally poor mental health or declining mental health during the same period.

What do you think makes the difference?

What is your impression of the mental health and wellbeing of older women in Australia?

Prompt: What role do you think might be played by:

Physical activity?

Nutrition?

Difficult events in women's lives?

Stress?

Social support? Loneliness or isolation?

Financial security?

Illness?

Disability?

Mistreatment by partners, family members, or others? Includes emotional, financial, physical.

Personal experience

I'd now like to ask you about your own experience of wellbeing and mental health.

What can you tell me about it?

What do you do that gives you pleasure?

What makes you feel valued?

What undermines or threatens your mental health?

Advice

What advice would you like to give to women about caring for their mental health as they grow older?

What advice do you have for those who care for older women?

Prompt: Partners and families?
Healthcare providers such as GPs?
Organisations?
Governments?

What resources do you think would be useful to help women to improve and maintain mental health?

Demographic information

We're almost at the end now. Thank you very much for being so generous with your time.

We'd like to be able to give a summary of all the women who have talked to us, so may I check some facts with you? None of these will be used to identify you; it's just so that we can describe things like women's average age, and where they come from. Some of these things you've told me already, but I might mention them again just to ensure I've noted the details correctly. *[NB Don't ask about what is very clear from the interview.]*

In what year were you born?

In what country were you born?

How would you describe your cultural background? *(Prompt if necessary. For example, are you: Aboriginal, Torres Strait Islander, Greek heritage, Vietnamese, Anglo?)*

What is your highest level of education? Primary school, secondary school, trade certificate, diploma, bachelor's degree, postgraduate qualification.

What is your occupation? If "Retired": What did you do before you retired?

If not already clear: Are you in paid employment at the moment? What do you do?

How would you describe your health in general?

Do you live in: the city? the suburbs? a regional town? a country area? a remote area?

Do you live alone or with others? If others, who lives with you?

How would you describe your home?

Owned or rented?

My own house? My own flat? Retirement village? Supported accommodation? Other?

Are there people who are dependent on you? Whom you help out?

Prompt, if necessary: Grandchildren? Children? Partner? Other family members? Neighbours? Volunteer work?

Do you have people who support you?

Prompt, if necessary: Partner? Children? Grandchildren? Other family members? Neighbours? Organisations?

TAKE GREAT CARE WITH PARTNER QUESTIONS; they might be a source of distress, including bereavement. If this information has already been revealed, do not ask again.

Do you have a partner? *If yes:* How long have you been together? Do you live together?

If no: Have you had a long-term partner?

What is/was your partner's gender?

<u>**Anything we've missed?**</u>

That's all I have to ask you.

Is there anything else you'd like to tell us about how to improve or maintain women's mental health and wellbeing?

[Thank the participant for her generous contribution to the research.]
